# A Loss of Function Screen of Epigenetic Modifiers and Splicing Factors during Early Stage of Cardiac Reprogramming

**DOI:** 10.1155/2018/3814747

**Published:** 2018-03-18

**Authors:** Yang Zhou, Sahar Alimohamadi, Li Wang, Ziqing Liu, Joseph B. Wall, Chaoying Yin, Jiandong Liu, Li Qian

**Affiliations:** ^1^Department of Pathology and Laboratory Medicine, University of North Carolina, Chapel Hill, NC 27599, USA; ^2^McAllister Heart Institute, University of North Carolina, Chapel Hill, NC 27599, USA

## Abstract

Direct reprogramming of cardiac fibroblasts (CFs) to induced cardiomyocytes (iCMs) is a newly emerged promising approach for cardiac regeneration, disease modeling, and drug discovery. However, its potential has been drastically limited due to the low reprogramming efficiency and largely unknown underlying molecular mechanisms. We have previously screened and identified epigenetic factors related to histone modification during iCM reprogramming. Here, we used shRNAs targeting an additional battery of epigenetic factors involved in chromatin remodeling and RNA splicing factors to further identify inhibitors and facilitators of direct cardiac reprogramming. Knockdown of RNA splicing factors Sf3a1 or Sf3b1 significantly reduced the percentage and total number of cardiac marker positive iCMs accompanied with generally repressed gene expression. Removal of another RNA splicing factor Zrsr2 promoted the acquisition of CM molecular features in CFs and mouse embryonic fibroblasts (MEFs) at both protein and mRNA levels. Moreover, a consistent increase of reprogramming efficiency was observed in CFs and MEFs treated with shRNAs targeting Bcor (component of BCOR complex superfamily) or Stag2 (component of cohesin complex). Our work thus reveals several additional epigenetic and splicing factors that are either inhibitory to or required for iCM reprogramming and highlights the importance of epigenetic regulation and RNA splicing process during cell fate conversion.

## 1. Introduction

Mammalian hearts have limited ability to regenerate, thus deleterious insult such as myocardial infarction (MI) can result in a permanent loss of cardiomyocytes (CMs) and a progressive decline in heart function [1]. So far, there is limited treatment to fully restore heart function after cardiac injury, ultimately leading to heart failure that becomes the leading cause of death worldwide. Recently, several promising strategies emerged to replenish the lost endogenous CMs or replace the malfunctioning CMs, including the ones using autologous sources of CMs derived from cardiac progenitor/stem cells, pluripotent stem cell, or directly induced cardiomyocytes (iCMs) [2]. Among them, direct reprogramming of fibroblasts into iCMs has been vigorously pursued in recent years, because of its feasibility both in vitro and in vivo and its unique process without passing through a pluripotent or progenitor stage, which can potentially avoid the risk of tumorigenesis. It was first reported that three master transcription factors, Gata4, Mef2c, and Tbx5, are capable of directly converting mouse cardiac fibroblasts (CFs) into iCMs in vitro [3]. Subsequently, generation of iCMs in vivo became possible in a murine MI model, resulting in functional improvement and scar size reduction [4, 5]. Thereafter, a growing number of studies have been performed focusing on alternative cocktails that could improve efficiency and/or purity of iCMs [4, 6–16] and began to reveal the underlying molecular mechanisms during iCM reprogramming [17–22]. Despite these advances, the potential of iCM approach to be used on patients is still limited because of the relatively low efficiency and largely unknown molecular mechanisms, which have to be fully elucidated before future clinical implementation.

Epigenetics is defined as stable and heritable changes in gene expression or cellular phenotype that does not involve changes in DNA sequence [23, 24]. Although the cell fate conversion requires instructive cues via ectopic expression of master transcription factors, the successful reprogramming relies on and can be greatly enhanced by epigenetic modification that is necessary for establishing and maintaining altered gene expression patterns over rounds of cell division. As such, epigenetic regulation is critical for cellular reprogramming as elaborated in other direct reprogramming processes [25]. We and others have shown that repatterning of H3K27me3, H3K4me3, and DNA methylation is accompanied with alternation in gene transcription during early stage of cardiac reprogramming from fibroblasts [3, 17, 19, 26], and removal of epigenetic barriers associated with histone modifications, such as Bmi1 and Mll1, significantly improved quantity and quality of iCMs [18, 21]. However, besides histone modifications and DNA methylation, the epigenetic processes that stably sustain gene expression also include chromatin remodeling and various RNA-mediated processes, and the role of the related epigenetic regulators remains largely unknown in direct cardiac reprogramming. Recent studies on heart development and cellular reprogramming demonstrated that the coordination of transcription factors and chromatin remodeling is critical for cell fate determination and conversion [25, 27, 28]. Therefore, despite what has been studied, it is important to identify key chromatin remodeling-related epigenetic regulators that orchestrate iCM induction. Characterization of each epigenetic modulator will help understand how cells with identical DNA reprogrammed into different lineages and delineate the role of epigenetic barriers and facilitators involved in not only iCM reprogramming but also maybe other cellular reprogramming processes.

RNA splicing is increasingly being recognized as an important layer of posttranslational gene regulation in the heart [29]. For instance, splicing factor Sf3b1, a component of U2 snRNPS involved in both constitutive and alternative splicing, is dysregulated in human and mouse models of pathological cardiac hypertrophy [30]. Moreover, reversion of global splicing pattern has been demonstrated to occur during somatic cell reprogramming [31]. Removal of splicing factors U2af1 and Srsf3 decreased reprogramming efficiency of induced pluripotent stem cells (iPSCs) [31]. Of note, we recently found that removal of alternative splicing factor Ptbp1 significantly promoted cardiac fate conversion from fibroblasts [22]. These studies highlighted the regulation of RNA splicing as part of the mechanisms underlying cellular reprogramming and pathogenesis of heart disease and implied the potential key role of RNA splicing factors for iCM reprogramming. Thus, identifying functional splicing factors during direct cardiac reprogramming will provide further insight into our understanding of the molecular mechanisms underlying iCM induction.

Here, we screened epigenetic modulators associated with different complexes and core splicing factors by shRNA-mediated loss of function assays and identified splicing factors Sf3a1 and Sf3b1 that are required for direct cardiac reprogramming. Meanwhile, the removal of another splicing factor Zrsr2 enhanced generation of iCMs from CFs and MEFs. Additional two epigenetic regulators, Bcor and Stag2, were implicated as independent epigenetic inhibitors to iCM reprogramming. These findings provide additional insights into the critical roles of epigenetic modulators and splicing factors on direct cardiac lineage conversion and the basis for future further investigation of epigenetic and RNA splicing-related mechanisms underlying reprogramming.

## 2. Materials and Methods

### 2.1. Mouse Lines

The transgenic mice carrying *α*MHC-GFP reporter were used for isolation of cardiac fibroblasts (CFs) and mouse embryonic fibroblasts (MEFs) [3, 5]. All mouse protocols were approved by the Institutional Animal Care and Use Committee (IACUC), University of North Carolina, Chapel Hill. Animal care was performed in accordance with the guidelines established by the University of North Carolina, Chapel Hill.

### 2.2. Plasmids

The polycistronic construct pMXs-puro-MGT was constructed as previously described [14]. The plasmid map of pMXs-puro-MGT is provided in Figure S1. shRNA lentiviral vectors with pLKO.1 backbone were obtained from Sigma-Aldrich. Packaging and envelop vectors for lentivirus were psPAX2 and pMD2.G (Addgene).

### 2.3. Virus Packaging and Transduction

PlatE cells were cultured in 293T media (10% fetal bovine serum (FBS)/1x penicillin/streptomycin (P/S)/0.1 mM nonessential amino acids (NEAA)/DMEM) (Life Technologies). Four to five million platE cells seeded onto a 10 cm dish were used for transfection. The next day, pMXs-puro-MGT were introduced into platE cells using Nanofect (Alstem) according to manufacturer's instructions. Briefly, 20 *μ*g of pMXs-puro-MGT and 45 *μ*l of Nanofect were mixed with 500 *μ*l of DMEM in separate tubes, and the mixture was combined and vortexed for a few seconds. After 15 minutes of incubation at room temperature, 1 ml of total mixture was added dropwise to platE cells. Fresh 293T medium without P/S was replaced before transfection. 16 hours posttransfection, media were changed with regular 293T media. Supernatant containing retroviruses was collected 48 and 72 hours after transfection, filtered through a 0.45 *μ*m filter (Thermo Scientific), and incubated with 8% of PEG6000 (Sigma) at 4°C overnight. Viral particles were pelleted with centrifuge at 3900 rpm for 30 minutes at 4°C and resuspended with 100 *μ*l of DMEM. For CFs or MEFs in one well of 24 well plate, 10 *μ*l of pMX-puro-MGT supplemented with 4 *μ*g/ml polybrene (Life Technologies) was added for cardiac reprogramming.

Five million 293T cells seeded and cultured overnight in one 10 cm dish with 293T media were used for lentiviral packaging. 10 *μ*g of pLKO.1 mixture with shRNAs (equal amount) targeting one gene, 7 *μ*g of psPAX2, and 3 *μ*g of pMD2.G were mixed in 500 *μ*l of DMEM. The DNA mixture was combined with transfection reagent mixture containing 45 *μ*l of Nanofect and 500 *μ*l of DMEM, vortexed, and incubated at room temperature for 20 minutes. After media exchange 16 hours posttransfection, virus-containing media were collected at 48 and 72 hours posttransfection. Filtered media were incubated with 8% PEG6000 overnight at 4°C and centrifuged at 3900 rpm, 30 minutes at 4°C to obtain viral particles. 100 *μ*l of DMEM was used to resuspend viral particles. 10 *μ*l of lentiviruses was used for cells seeded on each well of 24-well plate coinfected with MGT retroviruses.

### 2.4. Isolation of CFs

Explant and fresh isolation of CFs were performed according to the protocols described as previously [14, 32]. Briefly, hearts were dissected from postnatal 1.5 (p1.5) mice, rinsed with cold PBS, and cut into small pieces with a sterile blade. For explant CFs, small tissues were plated onto gelatin-coated dishes and cultured in fibroblast (FB) media (IMDM/20% FBS/1xPen/Strep) for 7 days. Before magnetic-activated cell sorting (MACS), explanted heart cells were trypsinized and filtered through 40 *μ*m cell strainer (BD). To isolate fresh CFs, heart tissues were digested with 0.05% trypsin at 37°C for 10 minutes and 0.2% collagenase type II/HBSS (Life Technologies) at 37°C for 5 minutes followed by 1 minute of vortexing for 5 times. Each time, supernatant containing single cells was filtered through 40 *μ*m cell strainer (BD) and neutralized in equal volume of FB media. Red cells were removed using red cell lysis buffer (150 ml NH4Cl, 10 mM KHCO3, and 0.1 mM EDTA) for 1 minute on ice. Then, MACS was performed to enrich Thy1.2-positive fibroblasts. Cells were incubated with 10 *μ*l of biotin anti-Thy1.2 antibody (Biolegend) in FACS buffer (DPBS/2% FBS/2 mM EDTA) for 30 minutes at 4°C and then with 10 *μ*l of Anti-Biotin Microbeads (Miltenyi Biotec.) in MACS buffer (DPBS/0.5% BSA/2 mM EDTA) at 4°C for 30 minutes. After that, cells were washed and resuspended in MACS buffer and applied to calibrated LS column (Miltenyi Biotec.). Thy1.2-positive cells were flushed out and seeded for reprogramming.

### 2.5. Preparation of MEFs

MEFs were isolated from E13.5 *α*MHC-GFP pups from CD1 strain as previously described [33]. Briefly, embryos isolated from pregnant mice at E13.5 were dissected out heads and red organs and then mined with a sterile blade. Small tissues were dissociated in 1 ml of 0.05% trypsin/EDTA supplemented with 100 units of DNase I for 15 minutes at 37°C and then neutralized in MEF media (10%FBS/1XP/S/DMEM). After centrifuge, the cells were resuspended into MEF media and plated into gelatin-coated dishes. Then, MEFs with a low passage number (*n* = 2–6) were used for iCM reprogramming assays.

### 2.6. Direct Cardiac Reprogramming

**Figure 1:**
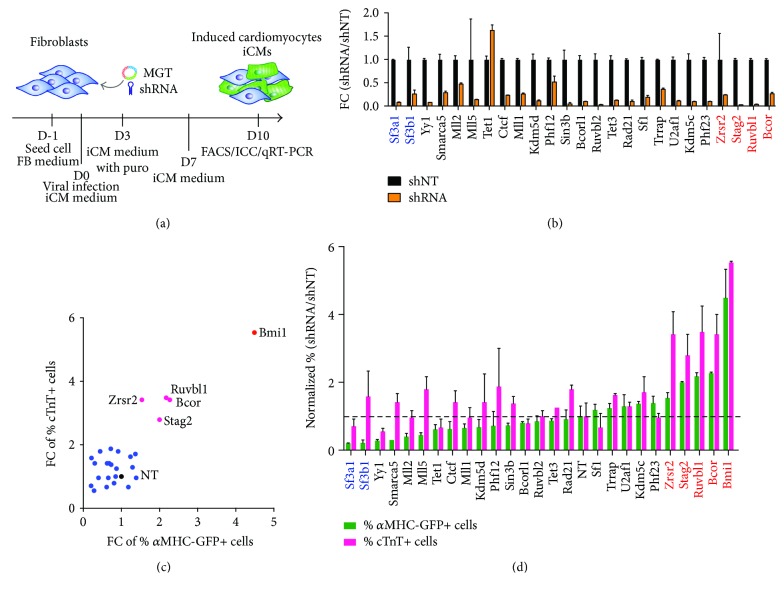
Knockdown of various epigenetic regulators influenced direct cardiac reprogramming. (a) Schematic of experimental design to determine the effect of candidate factors on iCM reprogramming via shRNA-mediated RNAi. FB stands for fibroblasts. (b) Knockdown efficiency of indicated shRNAs measured by RT-qPCR. Expression values were normalized to those measured in shNT-infected cells at reprogramming day 10. FC stands for fold change. (c) 25 selected chromatin modulators and splicing factors were knocked down in CFs coinfected with MGT for cardiac reprogramming. *α*MHC-GFP+ and cTnT+ cells were measured by flow cytometry at day 10 posttransduction. The percentage of marker positive cells was normalized to shNT-infected control cells shown in black. Bmi1 highlighted in red was used as a positive control for screening. (d) Histogram of normalized percentage of *α*MHC-GFP+ and cTnT+ cells after infection of MGT and shRNA lentiviruses targeting individual genes as indicated.

ExCFs, fCFs, and MEFs were seeded onto gelatin-coated wells of 24-well plates at a cell density of 2 × 10^4^ one day before infection. iCM media (10% FBS/20% M199/DMEM) with 10 *μ*l of retroviral puro-MGT, 10 *μ*l of lentiviral shRNAs, and 4 *μ*g/ml polybrene were replaced for FB media at reprogramming day 0. iCM media with 1 *μ*g/ml puromycin were used at day 3 and replaced by regular iCM media at day 6. At day 10, reprogramming cells were collected in TRIzol for RNA extraction or fixed with 4% paraformaldehyde (PFA) for immunostaining (Figure 1(a)).

### 2.7. Flow Cytometry and Immunocytochemistry (ICC)

For flow cytometry, reprogrammed cells were trypsinized with 0.05% trypsin/EDTA (Life Technologies), fixed with Fixation/Permeabilization Solution (BD Biosciences) for 30 minutes at 4°C. Perm/Wash Solution (BD Bioscience) was used for wash between each step. Cells were incubated with primary antibodies (GFP, 1 : 500, Invitrogen; cTnT, 1 : 400, Thermo Scientific) diluted in BD Perm/Wash Solution for 30 minutes at 4°C and Alex Fluor 488-conjugated or Alex Fluor 647-conjugated secondary antibodies (1 : 500, Jackson ImmunoResearch Inc.) for 30 minutes at 4°C. Cells were run on Beckman Coulter CyAn ADP flow cytometer. Data analyses were performed by FlowJo software (Tree Star). For ICC, 4% PFA-fixed cells were permeablized with 0.1% Triton-X100 for 20 minutes, blocked by 5% BSA for 30 minutes at room temperature, and then incubated with primary antibody (GFP, 1 : 500, Invitrogen; *α*Actinin, 1 : 500, Sigma-Aldrich; cTnT, 1 : 400, Thermo Scientific) at 4°C overnight and Alex Fluor 488-conjugated or Alex Fluor 647-conjugated secondary antibodies (1 : 500, Jackson ImmunoResearch Inc.) for 1 hour at room temperature. Finally, Hoechst 33342 (Life Technologies) was used to label nuclei. PBS was used for wash between each step. Images were captured using EVOS® FL Auto Cell Imaging System (Life Technologies). For the quantification of ICC, 10 images were randomly acquired under 20x magnification at the same exposure setting. Then the indicated cells were counted manually in a blind fashion.

### 2.8. RNA Extraction and Reverse Transcription Following Quantitative PCR (RT-qPCR)

According to the manufacturer's instruction, cell lysates in TRIzol reagent (Invitrogen) were separated with chloroform. RNA in the aqueous phase was precipitated with isopropanol, pelleted with centrifuge, washed with ethanol, and eluted in DNase-free and RNase-free water. Purified RNA was quantified by Nanodrop (Thermo Scientific) and reverse-transcribed into cDNA using SuperScript III Reverse Transcriptase (Invitrogen). qPCR was performed using Power SYBR Green PCR Master Mix (Applied Biosystems) on the ABI ViiA 7 Real-Time PCR system (Applied Biosystems). Additional primer sequences for RT-qPCR are provided in Supplemental Table S2.

### 2.9. Statistical Analyses

For each experiment, 3-4 biological replicates were examined with technical duplicates. Negative controls (i.e., mock-treated and/or nontargeting shRNA control transduced cells) and positive controls (i.e., shBmi1-transfected cells) were used in every single experiment. Average number from technical duplicates was used for statistics. For ICC, quantification was performed from 10 images randomly taken under 20x magnification at the same exposure setting in a blind fashion, and averaged numbers were used for final statistics. Where appropriate, values are presented as the mean ± SEM of replicate experiments. Statistical analyses were performed with two-way unpaired Student *t*-test or one-way ANOVA. ^∗^ indicates significant difference between two groups with a *p* value of *p* < 0.05, and ^∗∗^ *p* < 0.01, ^∗∗∗^ *p* < 0.001, and ^∗∗∗∗^ *p* < 0.0001 indicates highly significant difference.

## 3. Results

### 3.1. RNAi Screening for Epigenetic Regulators during Early Stage of Cardiac Reprogramming

We previously showed that removal of the key epigenetic barrier Bmi1 promotes the efficiency and quality of iCMs generated via transcription factor-mediated direct reprogramming [21]. In an effort to further examine the influence of additional epigenetic factors on iCM reprogramming, we used a similar loss-of-function screen to determine the role of 25 selected genes related to epigenetic modification and chromatin remodeling (Table 1). A pool of 4–6 short hairpins was used for knockdown of each gene (Table S1). We infected cardiac fibroblasts isolated using explant method (ExCFs, [3]) from *α*MHC-GFP transgenic pups at P1.5 with shRNA pools, subsequently transduced them with the polycistronic reprogramming vector expressing Mef2c, Gata4, and Tbx5 (MGT in short). We then determined the percentage of reprogrammed iCMs expressing *α*MHC-GFP and cardiac troponin T (cTnT) by flow cytometry (Figure 1(a)). Nontargeting shRNA and oligo-targeting Bmi1 were served as the negative and positive control, respectively [21]. After 10 days of infection, knockdown efficiency of shRNAs was first determined by real-time quantitative PCR (RT-qPCR) (Figure 1(b)). Then, generation of iCMs upon knockdown of various genes was scored by the fold changes relative to shNT control in the percentage of *α*MHC-GFP+ cells and cTnT+ cells. We found that knockdown of four genes showed a significant increase in reprogramming efficiency when compared to control shNT, but still lower than that when Bmi1 was knocked down (Figure 1(c)). Among the target genes are BCL6-interacting corepressor (*Bcor*) and RuvB-like protein 1(*Ruvbl1*), two chromatin modifiers studied in various chromatin complexes [34–38]. The remaining two shRNAs targeted one of the components of cohesin complex, stromal antigen 2 (*Stag2*), and one of the splicing factors for spliceosome assembly, zinc finger (CCCH type) RNA binding motif, and serine/arginine rich 2 (*Zrsr2*). On the contrary, knockdown of several members of SET1/MLL protein family, lysine- (K-) specific methyltransferase 2A (*Mll1/Kmt2a*), lysine- (K-) specific methyltransferase 2D (*Mll2/Kmt2d*), and lysine- (K-) specific methyltransferase 2E (*Mll5/Kmt2e*), showed repressive effects on generation of *α*MHC-GFP+ iCMs, suggesting the essential roles of H3K4 methylation for cardiac reprogramming (Figure 1(d)). Interestingly, the top targets, knocking down of which caused 5-fold decreases in the percentage of *α*MHC-GFP+ iCMs, are splicing factor 3a, subunit 1 (*Sf3a1*), and splicing factor 3b, subunit 1 (*Sf3b1*). Both belong to splicing factors of spliceosome similarly as Zrsr2 but have opposite phenotypes upon depletion compared to deletion of Zrsr2. Taking advantage of shRNA-mediated RNAi screen, we identified various epigenetic modulators that are either required for or inhibitory to iCM reprogramming.

### 3.2. Impaired Cardiac Reprogramming after Knockdown of *Sf3a1* or *Sf3b1*

**Figure 2:**
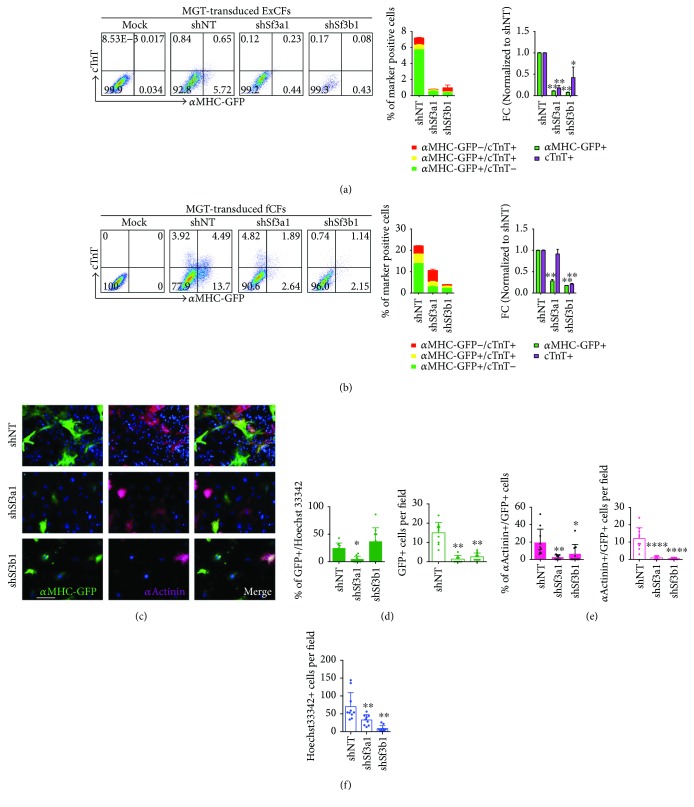
Knockdown of *Sf3a1* or *Sf3b1* inhibited the generation of iCMs. (a) Flow cytometry analysis (left) for *α*MHC-GFP+ and cTnT+ cells reprogrammed from ExCFs 10 days after infection of MGT with shNT, shSf3a1, or shSf3b1. The histograms (right) showed percentage and normalized fold change of *α*MHC-GFP+ and/or cTnT+ cells measured by flow cytometry. (b) Flow cytometry analysis (left) for *α*MHC-GFP+ and cTnT+ cells reprogrammed from fCFs 10 days after infection of MGT with shNT, shSf3a1, or shSf3b1. The histogram (right) showed percentage and normalized fold change of *α*MHC-GFP+ and/or cTnT+ cells measured by flow cytometry. (c) Representative ICC images for *α*MHC-GFP+ and *α*Actinin+ cells on MGT-transduced ExCFs coinfected with shNT, shSf3a1, or shSf3b1. Scale bar, 100 *μ*m. (d) ICC quantification of percentage and cell number of *α*MHC-GFP+ cells indicated in (c). (e) ICC quantification of percentage and cell number of cells expressing both *α*MHC-GFP+ and *α*Actinin+ in (c) samples. (f) Total cell number of cells in (c) labeled by Hoechst 33342. ^∗^ *p* < 0.05, ^∗∗^ *p* < 0.01, and ^∗∗∗∗^ *p* < 0.0001.

Spliceosome is a complex and highly dynamic molecular machinery that recognizes the splice sites and removes introns from precursor messenger RNAs (pre-mRNAs) [39, 40]. Five small ribonuclear protein particles (snRNPs) and various accessory proteins are assembled to form the spliceosome [39, 40]. Sf3a1 and Sf3b1 are components of U2 snRNP, which stabilizes U2 snRNA binding to the branch point sequence in introns [41, 42]. The mutations of these two splicing factors are found to be associated with myelodysplastic syndrome [43]. However, it is still largely unknown about the cellular functions of Sf3a1 or Sf3b1. Through our shRNA screen, we are intrigued to find that knockdown of two splicing factors involved in spliceosome assembly led to inhibition of iCM generation. We then confirmed shRNA screen results by both flow cytometry and quantification of iCMs generated from ExCFs. At day 10 after infection of shSf3a1 or shSf3b1 lentiviruses on MGT-transduced ExCFs, the percentage of cells expressing cardiac marker, *α*MHC-GFP, or cTnT and positive cells dropped dramatically (Figure 2(a)). To further confirm the essential roles of Sf3a1 and Sf3b1 for iCM reprogramming, we performed knockdown experiments on freshly isolated CFs (fCFs), which seems most amenable for MGT-mediated cardiac reprogramming [21], and assessed reprogramming efficiency by flow and ICC analyses of iCM-expressing cardiac markers. Consistently, knockdown of *Sf3a1* or *Sf3b1* repressed the generation of *α*MHC-GFP and/or cTnT-positive iCMs derived from fCFs when compared to those from control shNT-infected CFs (Figure 2(b)). ICC images and quantifications showed similar decrease of reprogrammed cells expressing *α*MHC-GFP reporter and cardiac Z-disc protein, *α*Actinin upon knockdown of *Sf3a1* or *Sf3b1* (Figures 2(c)–2(e)). Meanwhile, we found that knockdown of these two splicing factors during reprogramming resulted in a significantly reduced total cell number indicated by Hoechst 33342 staining (Figure 2(f)). These data suggest that depletion of splicing factors not only decreased iCM reprogramming efficiency but also influenced cell survival under such context.

### 3.3. Enhanced Conversion of iCMs from CFs upon Knockdown of *Ruvbl1*, *Bcor*, *Zrsr2*, or *Stag2*

**Figure 3:**
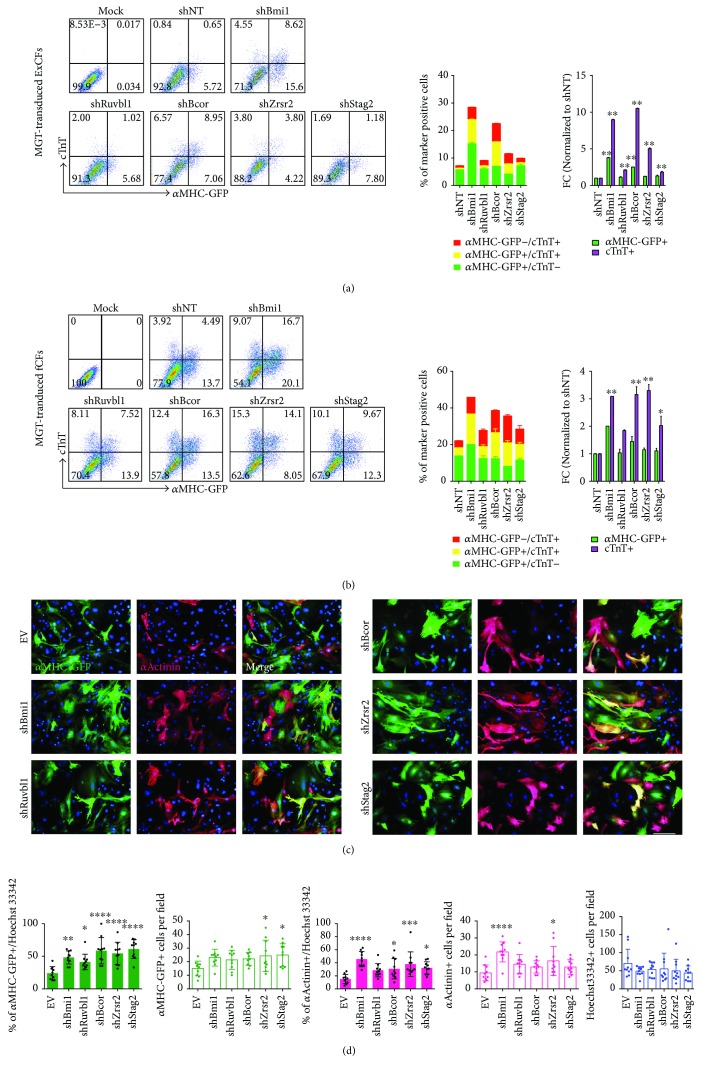
Knockdown of *Ruvbl1*/*Bcor*/*Zrsr2*/*Stag2* enhanced the efficiency of cardiac reprogramming from CFs. (a) Representative flow plots (left) with quantification (right) for *α*MHC-GFP+ and cTnT+ cells derived from ExCFs 10 days postinfection of MGT and shRNA lentiviruses as indicated. (b) Representative flow plots (left) with quantification (right) for *α*MHC-GFP+ and cTnT+ cells derived from fCFs 10 days postinfection of MGT and shRNA lentiviruses as indicated. (c) Representative images of ICC for *α*MHC-GFP+ and *α*Actinin+ cells derived from fCFs after infection of MGT and indicated shRNAs at reprogramming day 10. (d) ICC quantification for *α*MHC-GFP+ and *α*Actinin+ cells and total cells indicated by Hoechst 33342 in (c). ^∗^ *p* < 0.05, ^∗∗^ *p* < 0.01, ^∗∗∗^ *p* < 0.001, and ^∗∗∗∗^ *p* < 0.0001.

On the other hand, we further confirmed the phenotypes resulted from knockdown of the top four hits, *Ruvbl1*, *Bcor*, *Zrsr2*, and *Stag2* that seem to be inhibitors of direct cardiac reprogramming. Ruvbl1 (or Tip48, potin) belongs to the AAA+ ATPase (ATPase associated with multiple activities) family [36]. A number of chromatin-remodeling complexes contain Ruvbl1, like INO80 complex, TIP60 complex, and SWR1 complex, to facilitate the assembling and maintenance of the catalytic activity of ATPase [36, 38, 44]. It has been reported that INO80 complex is required for embryonic stem cell self-renewal and pluripotency via exchange and deposition of histone variant H2A.Z [45]. Here, to further confirm the role of Ruvbl1 as the potential inhibitor for cardiac reprogramming, we assessed iCM generation from ExCFs and fCFs by flow cytometry and ICC staining. shNT was used as a negative control, while shBmi1 as a positive control. Flow cytometry results showed a 2-fold increase in the percentage of cTnT+ iCMs from MGT-transduced ExCFs and fCFs after infection of shRuvbl1 lentiviruses (Figures 3(a) and 3(b)). In addition, loss of *Ruvbl1* led to a significant increase in the percentage of *α*MHC-GFP+ cells derived from fCFs (Figures 3(c) and 3(d)). Interestingly, knockdown of the other RVB gene Ruvbl2 did not appear to affect the reprogramming efficiency (Figure 1(d)), indicating distinct roles of different RVB genes during conversion from fibroblasts to iCMs.

Bcor has been identified as a transcriptional corepressor and known to regulate gene expression in association with epigenetic-modifying complexes including Polycomb group (PcG) proteins, Skp-Cullin-F-box (SCF) ubiquitin ligase, and histone demethylase [34, 35]. *Bcor* is a ubiquitously expressed gene and related to X-linked oculofaciocardiodental (OFCD) syndrome exemplified by multiple defects in human, such as cardiac atrial septal defect [34, 46]. Study of Bcor loss of function mutant mice showed a strong parent-of-origin effect, indicating a possible regulatory role of Bcor in extraembryonic tissues during early development [46]. Moreover, Bcor is required for proper differentiation of embryonic stem cells into ectoderm, mesoderm, and downstream hematopoietic lineages [46]. This is the first time that *Bcor* was investigated in iCM reprogramming. We found that knockdown of *Bcor* resulted in 10-fold and 3-fold increases in percentage of cTnT+ iCMs derived from ExCFs and fCFs, respectively, when compared to treatment of shNT (Figures 3(a) and 3(b)). Increased percentages of additional cardiac markers *α*MHC-GFP and *α*Actinin-positive iCMs upon *Bcor* knockdown were confirmed by ICC staining (Figures 3(c) and 3(d)). Notably, based on cardiac marker expression measured by flow and ICC, knockdown of *Bcor* resulted in the highest increase in reprogramming efficiency among the top four candidates.

Zrsr2 (a homolog of U2AF35) is another essential component of the spliceosome [47]. Zrsr2 (or Urp) physically interacts with U2AF65 and serine/arginine-rich (SR) proteins to facilitate the recognition of exon/intron boundary and spliceosome assembly [47, 48]. After 10 days of MGT transduction, a significant increase in the percentage of cTnT+ cells was detected under treatment of shZrsr2 in both ExCFs and fCFs when compared to that obtained with control shNT treatment (Figures 3(a) and 3(b)). Moreover, the percentage as well as the absolute number of *α*MHC-GFP+ or *α*Actinin+ cells significantly increased upon the removal of *Zrsr2* (Figures 3(c) and 3(d)). Taken together, our data indicated that the loss of function of *Zrsr2* enhanced conversion from cardiac fibroblasts to iCMs.

Stag2 (or SA2) encodes one of the core subunits of cohesin complex, which holds sister chromatids in dividing cells and is essential for chromatin segregation [49, 50]. In addition, cohesin has been recently implicated in chromatin looping and insulation via its direct interaction with CTCF to control chromatin structure and gene regulation [51–53]. Similarly, independent of different isolation methods to prepare the starting CFs, loss of *Stag2* always led to significant increases in both percentage and number of iCMs as shown by flow cytometry for *α*MHC-GFP+/cTnT+ cells and ICC analysis of *α*MHC-GFP+ or *α*Actinin+ cells (Figures 3(a)–3(d)). However, knockdown of another component of cohesion complex *Rad21* did not affect iCM reprogramming (Figure 1(d)), suggesting the complexity of underlying mechanism by which Stag2 and associated cohesion complexes function during direct cardiac reprogramming.

### 3.4. Enhanced iCM Generation from MEFs Depleted with *Bcor*, *Zrsr2*, or *Stag2*

**Figure 4:**
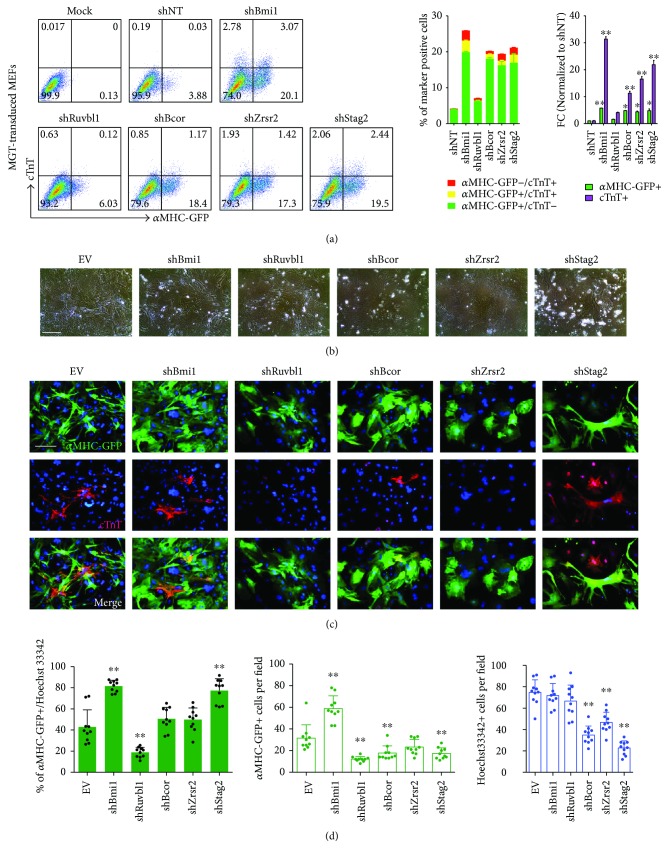
Knockdown of *Bcor*/Zrsr2/Stag2 promoted iCM conversion from MEFs. (a) Representative flow plots (left) and quantification (right) for *α*MHC-GFP+ and/or cTnT+ cells on MEFs after infection of MGT with indicated shRNA lentiviruses for 10 days. (b) Representative phase-contrast images of MGT-infected MEFs at day 10 after transduction of shRNAs as indicated. EV, empty vector, was used as a negative control. (c) Representative ICC images for *α*MHC-GFP or cTnT expressed iCMs derived from MEFs transduced with MGT and indicated shRNAs at reprogramming day 10. EV, empty vector, was used as a negative control. (d) Quantification for *α*MHC-GFP+ cells and total cells indicated by Hoechst 33342 in (c). ^∗^ *p* < 0.05, ^∗∗^ *p* < 0.01.

To rule out the cell type-specific roles of candidate epigenetic factors on cardiac reprogramming, we utilized mouse embryonic fibroblasts (MEFs) isolated from E13.5 pups from *α*MHC-GFP transgenic mice to test the effects of knockdown of *Ruvbl1*, *Bcor*, *Zrsr2*, and *Stag2*. After 10 days of MGT induction, *α*MHC-GFP+ and/or cTnT+ iCMs were assessed by flow cytometry on MEFs infected with shRNAs targeting *Ruvbl1*, *Bcor*, *Zrsr2*, *Stag2*, or control sequences (Figure 4(a)). Noticeably, knockdown of *Bcor*, *Zrsr2*, or *Stag2* resulted in about 5-fold increase in the percentage of *α*MHC-GFP+ iCMs when compared to shNT-treated cells (Figure 4(a)). However, knockdown of *Ruvbl1* led to merely 2-fold increase in the percentage of *α*MHC-GFP+ cells (Figure 4(a)). Generally, the fold change of the percentage of cTnT+ cells increased to various degree upon depletion of candidate four genes, but the percentage of cTnT+ cells derived from MEFs was always lower than that obtained from CFs (Figures 3(a) and 4(a)), suggesting the varied plasticity of fibroblasts from different origin with cardiac fibroblasts being most amenable. Interestingly, the bright field cell images showed that MGT-transduced cells became flat after knockdown of *Bcor* or *Zrsr2* and extensive cell death was observed from shStag2-infected MEFs (Figure 4(b)), confirmed by Hoechst 33342 staining (Figure 4, third graph). However, the total cell number when reprogramming fCFs was unaltered (Figure 3(d), fifth graph), suggesting that starting fibroblasts with diverse origins responded differently upon loss of epigenetic factors involved in a wide spectrum of chromatin regulatory complexes. Moreover, we performed ICC staining of cardiac reporter *α*MHC-GFP and cTnT on MGT-transduced MEFs treated with shRNAs targeting candidate genes. Noticeably, the percentage of *α*MHC-GFP+ cells was only significantly increased in shStag2-treated (2-fold increase) MEFs when compared to that in shNT-treated cells (Figures 4(c) and 4(d)). However, the absolute number of not only *α*MHC-GFP+ iCMs but also total cells was reduced after knockdown of *Bcor*/*Zrsr2*/*Stag2* (Figure 4(d)), suggesting the influence of epigenetic disruption on basic cell survival and growth of MEFs. Meanwhile, we found that loss of *Ruvbl1* did not affect iCM reprogramming efficiency from MEFs at day 10 upon transduction of MGT (Figures 4(a), 4(c), and 4(d)), indicating that the repressive function of Ruvbl1 might be CF specific and suggesting the potential variability of epigenetic status among multiple cell types. Taken these data together, we suggest Bcor/Zrsr2/Stag2 as inhibitory epigenetic regulatory factors during cardiac reprogramming from several fibroblast types.

### 3.5. Gene Expression Analyses of Reprogramming Cells after Knockdown of Epigenetic and Splicing Factors

**Figure 5:**
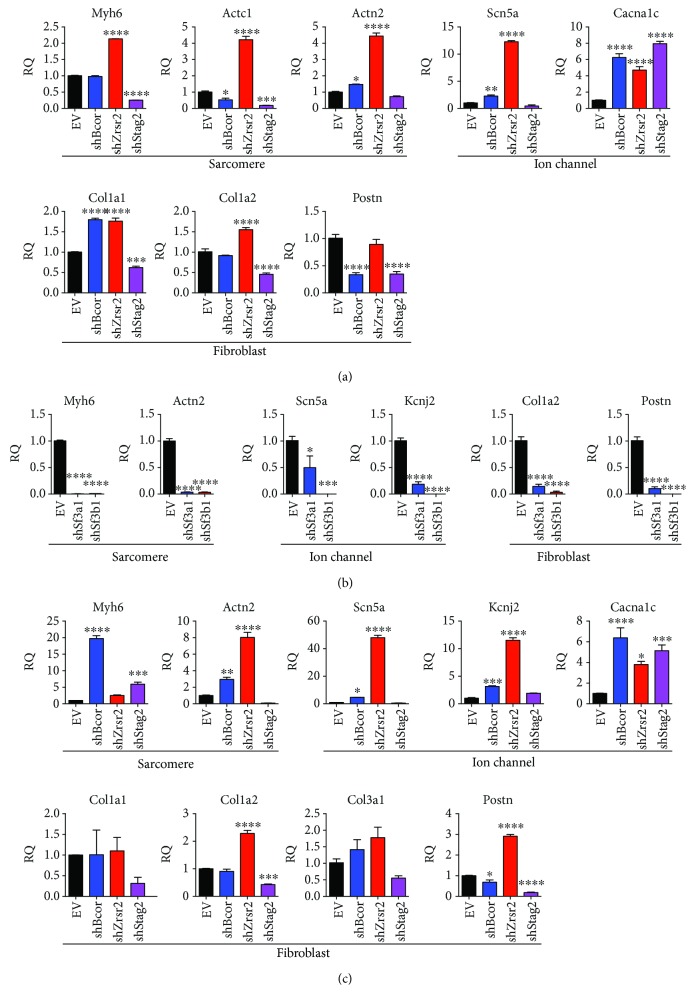
Knockdown of epigenetic factors changed the molecular features of reprogrammed cells. (a) Relative expression of cardiomyocyte-related sarcomere and ion channel genes or fibroblast marker genes in ExCFs infected with MGT and EV, shBcor, shZrsr2, or shStag2 at 10 days after infections. EV, empty vector, was used as a negative control. (b) Relative expression of sarcomere, ion channel, and fibroblast marker genes in MGT-infected ExCF at day 10 postinfection of EV, shSf3a1, or shSf3b1. EV, empty vector, was used as a negative control. ^∗^ *p* < 0.05, ^∗∗∗^ *p* < 0.001, and ^∗∗∗∗^ *p* < 0.0001. (c) Relative expression of cardiomyocyte-related sarcomere and ion channel genes or fibroblast marker genes in MEFs infected with MGT and indicated shRNAs at 10 days after infections. EV, empty vector, was used as a negative control. ^∗^ *p* < 0.05, ^∗∗^ *p* < 0.01, ^∗∗∗^ *p* < 0.001, and ^∗∗∗∗^ *p* < 0.0001.

To further explore how knockdown of epigenetic and splicing regulators influenced the expression profile of iCMs, we performed RT-qPCR with a set of CM marker genes related to sarcomere structure formation, ion channel, and fibroblast marker genes in reprogramming ExCFs coinfected with shRNA lentiviruses. Interestingly, loss of *Zrsr2* resulted in the highest increase in cardiac marker expression yet with no change in fibroblast marker expression (Figure 5(a)). Meanwhile, knockdown of *Stag2* significantly repressed fibroblast gene expression, as well as the expression of cardiac genes (Figure 5(a)), suggesting the essential role of Stag2 on global gene expression. On the other hand, depletion of core spliceosome factor *Sf3a1* and *Sf3b1* seems generally interfered with expression of all marker genes regardless of cell lineage. A dramatic decrease of these maker genes was found under treatment of shSf3a1 or shSf3b1 (Figure 5(b)), suggesting the essential role of U2-dependent spliceosome in maintaining gene expression in fibroblasts. Moreover, similar phenotypes after knockdown of *Bcor*/*Zrsr2*/*Stag2* were obtained by RT-qPCR of the same marker genes in MGT-transduced MEFs (Figure 5(c)). Therefore, we discovered gene expression patterns of reprogramming cells resulted from knockdown of distinct epigenetic factors, indicating the varied molecular response and potential underlying mechanisms upon manipulation of different epigenetic complexes or splicing factors that potentially orchestrate the expression of cardiac and fibroblast-related genes.

## 4. Discussion

In this study, we performed a shRNA-mediated loss of function screen for epigenetic modulators involved in chromatin remodeling and RNA splicing factors during direct cardiac reprogramming. We demonstrated that splicing factors Sf3a1 and Sf3b1 are required for cardiac reprogramming, while Zrsr2 is inhibitory to iCM induction. Moreover, we found that removal of *Bco*r and *Stag2* increased reprogramming efficiency regardless of the origins of starting fibroblasts, indicating that Bcor-related BCOR complex and Stag2-involved cohesin complex may play suppressive roles during conversion of iCMs. Although the detailed mechanisms by which these factors orchestrate iCM reprogramming remain to be elucidated, our results reveal additional regulators participating in the molecular networks underlying direct conversion from fibroblasts to iCMs.

Taken together with our previous finding, splicing factors have been demonstrated to play critical roles during direct cardiac reprogramming [22]. Knockdown of *Sf3a1* and *Sf3b1*, which are core components of U2 snRNP assembling U2-dependent major spliceosome, drastically reduced total reprogramming cell number and suppressed the mRNA level of both cardiac and fibroblast genes. However, knockdown of *Zrsr2*, which not only participates in the U2-dependent major splicing but is also required for U12-dependent minor splicing [48], enhanced cardiac reprogramming with increased iCM percentage and gene expression of cardiac markers. The opposite phenotypes might be associated with the complexity and dynamics of spliceosome and cell type-specific function of splicing factors. Likewise, we observed different effects of U2af1 knockdown on iCM generation from that on iPSC reprogramming [31], suggesting distinct RNA splicing regulation between iCM and iPSC reprogramming processes.

Although cohesin has been widely considered to be required for ESC self-renewal and identified as facilitators of iPSC reprogramming [53–55], it is intriguing to find that knockdown of the core component of cohesin *Stag2* facilitates iCM generation accompanied with decreased expression of fibroblast markers, suggesting cohesin as a potential barrier to direct cardiac reprogramming. In addition, cohesin-depleted ESCs and iPSCs are difficult to maintain or establish pluripotency gene expression, which could be explained by a loss of long-range interactions [54, 55] or by DNA damage responses resulted from defects in proliferation [56]. However, studies that excluded the influence of cell proliferation demonstrated that cohesin depletion enhanced the ability of ES cells to initiate somatic cell reprogramming [57]. Likely, in our study, the nondividing features of fully reprogrammed iCMs might partially explain the opposite roles of cohesin in iCM versus iPSC reprogramming. Additionally, cohesin also contributes to the establishment and maintenance of tissue-specific gene expression [52, 58]. Therefore, it will be particularly interesting to investigate cell type-specific role of cohesin in gene regulation during different cell fate conversion processes.

## 5. Conclusions

In this study, we employed shRNA-mediated RNAi screen and identified splicing factors Sf3a1 and Sf3b1 as essential regulators while splicing factor Zrsr2 and epigenetic modulators Bcor and Stag2 as inhibitory barriers for direct cardiac reprogramming. Our finding provides not only insights into understanding of molecular mechanisms of iCM reprogramming but also potential RNAi-based approach to improve reprogramming efficiency.

## Figures and Tables

**Table 1:** Categories and functional description of shRNA target genes.

shRNA target	Category	Description
Kdm5c/Jarid1c/Smcx	Epigenetic modulator	Histone H3 lysine 4 demethylase
Kdm5d/Jarid1d/Smcy	Epigenetic modulator	Histone H3 lysine 4 demethylase
Kmt2a/Mll1	Epigenetic modulator	Member of SET1/MLL complexes
Kmt2d/Mll2	Epigenetic modulator	Member of SET1/MLL complexes
Kmt2e/Mll5	Epigenetic modulator	Member of SET1/MLL complexes
Phf23	Epigenetic modulator	Redear of H3K4me3/2
Ctcf	Epigenetic modulator	Chromatin insulator
Bcorl1	Epigenetic modulator	Component of BCOR complex (subtype of PRC1, PRC1.1)
Bcor	Epigenetic modulator	Component of BCOR complex (subtype of PRC1, PRC1.1)
Rad21	Epigenetic modulator	Component of cohesin complex
Stag2/SA2	Epigenetic modulator	Component of cohesin complex
Phf12	Epigenetic modulator	Component of EMSY/KDM5A/SIN3B complex
Sin3b	Epigenetic modulator	Component of EMSY/KDM5A/SIN3B complex
Trrap	Epigenetic modulator	Component of TIP60 complex
Yy1	Epigenetic modulator	Component of INO80 complex
Ruvbl2/Tip48	Epigenetic modulator	Component of INO80 subfamily (INO80, TIP60, and SWR complexes)
Ruvbl1/Tip49	Epigenetic modulator	Component of INO80 subfamily (INO80, TIP60, and SWR complexes)
Smarca5	Epigenetic modulator	Component of ISWI complex (SWI/SNF)
Tet1	Epigenetic modulator	DNA hydroxymethyltransferase
Tet3	Epigenetic modulator	DNA hydroxymethyltransferase
Sf1	Splicing factor	Splicing factor for spliceosome assembly
Sf3a1/PRP21	Splicing factor	Splicing factor for spliceosome assembly (U2 snRNP)
Sf3b1	Splicing factor	Splicing factor for spliceosome assembly (U2 snRNP)
U2af1	Splicing factor	Splicing factor for spliceosome assembly
Zrsr2/URP	Splicing factor	Splicing factor for spliceosome assembly

## Supplementary Materials

Supplementary Materials Figure S1: plasmid map of pMXs-puro-MGT. Diagram of the polycistronic retroviral vector-expressing Mef2c, Gata4, and Tbx5, separated by P2A and T2A. Table S1: sequences of shRNA oligoes used for RNAi screen. Table S2: list of primers and Taqman probes.
