# Supplementary material for: A Loss of Function Screen of Epigenetic Modifiers and Splicing Factors during Early Stage of Cardiac Reprogramming
Source: Stem Cells Int. 2018 Mar 18;2018:3814747. doi: 10.1155/2018/3814747 (PMC5878887; doi:10.1155/2018/3814747)
Supplement: Supplementary Materials — Figure S1: plasmid map of pMXs-puro-MGT. Diagram of the polycistronic retroviral vector-expressing Mef2c, Gata4, and Tbx5, separated by P2A and T2A. Table S1: sequences of shRNA oligoes used for RNAi screen. Table S2: list of primers and Taqman probes. [file 3814747.f1.pdf]

Figure S1

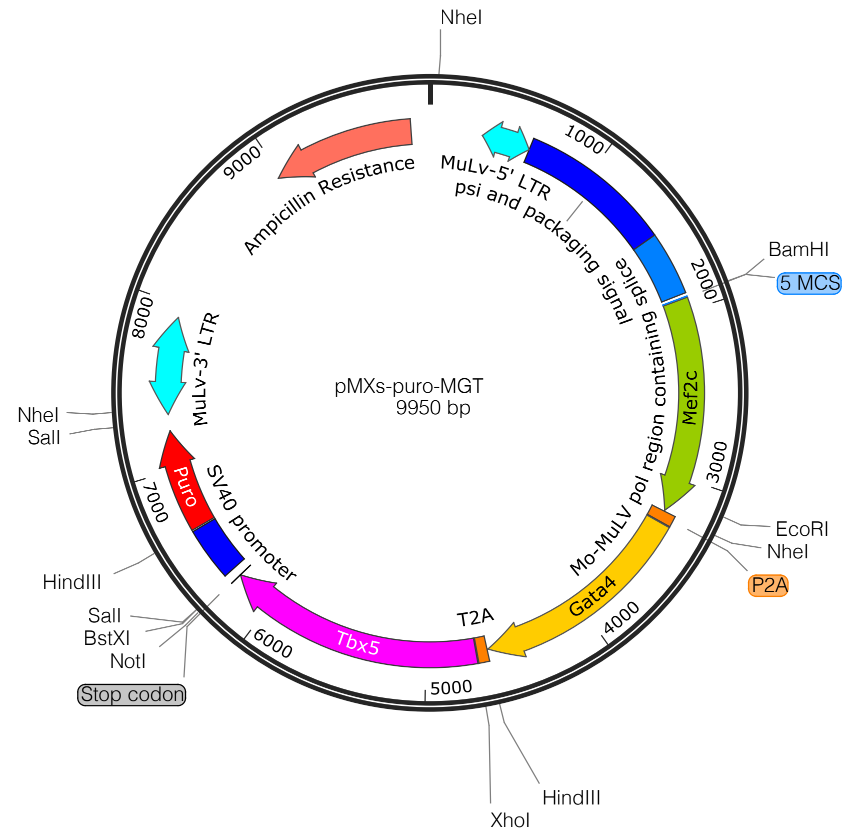
NheI
Ampicillin Resistance
9000
1000
MuLv-5' LTR
psi and packaging signal
splice
BamHI
5 MCS
2000
Mef2c
Mo-MuLV pol region containing
8000
MuLv-3' LTR
pMXs-puro-MGT
9950 bp
NheI
SalI
3000
Puro
SV40 promoter
7000
EcoRI
NheI
P2A
HindIII
Gata4
T2A
4000
SalI
BstXI
NotI
Stop codon
6000
Tbx5
5000
HindIII
XhoI

**Table S1. Sequences of shRNA oligoes used for RNAi screen.**

**Names and sequences of shRNAs cloned in pLKO.1 vetor**

**shRNA library for screen**

| Target Gene Symbol | TRC Id | Target Sequence (Sense) |
|---|---|---|
| shKdm5c_1 | TRCN0000295415 | CCGGAGCAAGCTACCCGGGAATATACTCGAGTATATTCCCGGGTAGCTTGCTTTTTG |
| shKdm5c_2 | TRCN0000295348 | CCGGATATTCTGACTCCAAGGTATTCTCGAGAATACCTTGGAGTCAGAATATTTTTG |
| shKdm5c_3 | TRCN0000287995 | CCGGCGCATTGTTTATCCCTATGAACTCGAGTTCATAGGGATAAACAATGCGTTTTTG |
| shKdm5c_4 | TRCN0000287921 | CCGGCGCTCTCACTATGAACGCATTCTCGAGAATGCGTTCATAGTGAGAGCGTTTTTG |
| shKdm5c_5 | TRCN0000287994 | CCGGGCCCAATATCCAGTCTCTCAACTCGAGTTGAGAGACTGGATATTGGGCTTTTTG |
| shKdm5c_6 | TRCN0000097857 | CCGGCGCATTGTTTATCCCTATGAACTCGAGTTCATAGGGATAAACAATGCGTTTTTG |
| shKdm5c_7 | TRCN0000097858 | CCGGCGCTCTCACTATGAACGCATTCTCGAGAATGCGTTCATAGTGAGAGCGTTTTTG |
| shKdm5c_8 | TRCN0000097859 | CCGGGCCCAATATCCAGTCTCTCAACTCGAGTTGAGAGACTGGATATTGGGCTTTTTG |
| shKdm5c_9 | TRCN0000097856 | CCGGGCATTGTTTATCCCTATGAAACTCGAGTTTCATAGGGATAAACAATGCTTTTTG |
| shKdm5d_1 | TRCN0000098105 | CCGGCCTAAGAAGTATTCCCTGTATCTCGAGATACAGGGAATACTTCTTAGGTTTTTG |
| shKdm5d_2 | TRCN0000098106 | CCGGCCTTTACTACACCTATCTTATCTCGAGATAAGATAGGTGTAGTAAAGGTTTTTG |
| shKdm5d_3 | TRCN0000098107 | CCGGGCAGAACACTTAGAGGATGTACTCGAGTACATCCTCTAAGTGTTCTGCTTTTTG |
| shKdm5d_4 | TRCN0000098108 | CCGGGCAATGTAACACAGATCCATTCTCGAGAATGGATCTGTGTTACATTGCTTTTTG |
| shKdm5d_5 | TRCN0000098109 | CCGGGCCTGTTATGACTGTCCAGATCTCGAGATCTGGACAGTCATAACAGGCTTTTTG |
| shMll1_1 | TRCN0000304329 | CCGGACGAAGATGACTTATACTATTCTCGAGAATAGTATAAGTCATCTTCGTTTTTTG |
| shMll1_2 | TRCN0000310900 | CCGGCTGATTCGCAAACCAATATTTCTCGAGAAATATTGGTTTGCGAATCAGTTTTTG |
| shMll1_3 | TRCN0000301690 | CCGGCGCCTTCACTTGACCATAATTCTCGAGAATTATGGTCAAGTGAAGGCGTTTTTG |
| shMll2_1 | TRCN0000239234 | CCGGGTTCATCGAGTTGCGACATAACTCGAGTTATGTCGCAACTCGATGAACTTTTTG |
| shMll2_2 | TRCN0000239233 | CCGGGACTGGTCTAGCCGATGTAAACTCGAGTTTACATCGGCTAGACCAGTCTTTTTG |
| shMll2_3 | TRCN0000239232 | CCGGCACCCACACCTACCTCATTTACTCGAGTAAATGAGGTAGGTGTGGGTGTTTTTG |
| shMll5_1 | TRCN0000241161 | CCGGACTCCAACTTCAATCACTTTACTCGAGTAAAGTGATTGAAGTTGGAGTTTTTTG |
| shMll5_2 | TRCN0000241164 | CCGGGCAACTTCTGGAGCGTTATTTCTCGAGAAATAACGCTCCAGAAGTTGCTTTTTG |
| shMll5_3 | TRCN0000241165 | CCGGATGCAGTGTTTGGCAACATATCTCGAGATATGTTGCCAAACACTGCATTTTTTG |
| shPhf23_1 | TRCN0000238013 | CCGGTGGGATCTGATCACGTGTTACCTCGAGGTAACACGTGATCAGATCCCATTTTTG |
| shPhf23_2 | TRCN0000238016 | CCGGGCGGAGAACCATTGAGGATTTCTCGAGAAATCCTCAATGGTTCTCCGCTTTTTG |
| shPhf23_3 | TRCN0000238017 | CCGGCCACTCTCTCCCACATCATTGCTCGAGCAATGATGTGGGAGAGAGTGGTTTTTG |
| shCtcf_1 | TRCN0000039019 | CCGGGCAGAGAAAGTAGTTGGTAATCTCGAGATTACCAACTACTTTCTCTGCTTTTTG |
| shCtcf_2 | TRCN0000039020 | CCGGCGGAACACAATGGCAAGACATCTCGAGATGTCTTGCCATTGTGTTCCGTTTTTG |
| shCtcf_3 | TRCN0000039021 | CCGGCCCATTAACATAGGAGAGCTTCTCGAGAAGCTCTCCTATGTTAATGGGTTTTTG |
| shCtcf_4 | TRCN0000039022 | CCGGCCGATGATATGTCACACCTTACTCGAGTAAGGTGTGACATATCATCGGTTTTTG |
| shCtcf_5 | TRCN0000039023 | CCGGGCCATCATTCAGGTCGAAGATCTCGAGATCTTCGACCTGAATGATGGCTTTTTG |
| shBcorl1_1 | TRCN0000085728 | CCGGGCCTTATCAATGCCAGCATTACTCGAGTAATGCTGGCATTGATAAGGCTTTTTG |
| shBcorl1_2 | TRCN0000085730 | CCGGGCACAGTGAAATCCCGTTATACTCGAGTATAACGGGATTTCACTGTGCTTTTTG |
| shBcorl1_3 | TRCN0000085731 | CCGGGCCACTCTAAGGAACTCATTTCTCGAGAAATGAGTTCCTTAGAGTGGCTTTTTG |
| shBcorl1_4 | TRCN0000085732 | CCGGGTGGTCAATGACAACCTAGAACTCGAGTTCTAGGTTGTCATTGACCACTTTTTG |
| shBcor_1 | TRCN0000081963 | CCGGCCCACTGTGTACAGTGTGTTACTCGAGTAACACACTGTACACAGTGGGTTTTTG |
| shBcor_2 | TRCN0000081965 | CCGGCCCGGATATTCCGTAGCAATTCTCGAGAATTGCTACGGAATATCCGGGTTTTTG |
| shBcor_3 | TRCN0000081966 | CCGGCCACTTAGAAATTGTACGATTCTCGAGAATCGTACAATTTCTAAGTGGTTTTTG |
| shBcor_4 | TRCN0000081967 | CCGGCCAGAATTTGTGACCTACCAACTCGAGTTGGTAGGTCACAAATTCTGGTTTTTG |
| shRad21_1 | TRCN0000175873 | CCGGGCACCAGAAATTATGTGACTACTCGAGTAGTCACATAATTTCTGGTGCTTTTTTG |
| shRad21_2 | TRCN0000175503 | CCGGCCATGTATTTGAGTGCAACTTCTCGAGAAGTTGCACTCAAATACATGGTTTTTTG |
| shRad21_3 | TRCN0000174832 | CCGGGCTTAGCGATTATTCTGATATCTCGAGATATCAGAATAATCGCTAAGCTTTTTTG |
| shRad21_4 | TRCN0000176084 | CCGGGATGAGTTCCTCAAAGAGTTTCTCGAGAAACTCTTTGAGGAACTCATCTTTTTTG |
| shRad21_5 | TRCN0000216543 | CCGGGCTTATAATGCCATTACTTTACTCGAGTAAAGTAATGGCATTATAAGCTTTTTTG |
| shStag2_1 | TRCN0000108975 | CCGGCGACCCTGAAATTGTAGGATACTCGAGTATCCTACAATTTCAGGGTCGTTTTTG |
| shStag2_2 | TRCN0000108976 | CCGGGCGCTTCTTGACCTTATCAATCTCGAGATTGATAAGGTCAAGAAGCGCTTTTTG |
| shStag2_3 | TRCN0000108977 | CCGGCCTCAACATTTAGTGGCATAACTCGAGTTATGCCACTAAATGTTGAGGTTTTTG |
| shStag2_4 | TRCN0000108978 | CCGGCCGATTTCTTTGATCCAGCTTCTCGAGAAGCTGGATCAAAGAAATCGGTTTTTG |
| shStag2_5 | TRCN0000108979 | CCGGCCTGATGAAGATGACGCATATCTCGAGATATGCGTCATCTTCATCAGGTTTTTG |
| shPhf12_1 | TRCN0000084419 | CCGGCCAACTCACTTCGAGCATTTACTCGAGTAAATGCTCGAAGTGAGTTGGTTTTTG |
| shPhf12_2 | TRCN0000084420 | CCGGCGGAGAAATCGAGATAAATATCTCGAGATATTTATCTCGATTTCTCCGTTTTTG |
| shPhf12_3 | TRCN0000084422 | CCGGCCTGAACCGAATCCACAAGAACTCGAGTTCTTGTGGATTCGGTTCAGGTTTTTG |
| shSin3b_1 | TRCN0000039367 | CCGGCCGTATAGACATTCCCAAGAACTCGAGTTCTTGGGAATGTCTATACGGTTTTTG |
| shSin3b_2 | TRCN0000287547 | CCGGCCGTATAGACATTCCCAAGAACTCGAGTTCTTGGGAATGTCTATACGGTTTTTG |

| | | |
|---|---|---|
| shYy1_1 | TRCN0000054553 | CCGGCGACGGTTGTAATAAGAAGTTCTCGAGAACTTCTTATTACAACCGTCGTTTTTG |
| shYy1_2 | TRCN0000054554 | CCGGCCCTAAGCAACTGGCAGAATTCTCGAGAATTCTGCCAGTTGCTTAGGGTTTTTG |
| shYy1_3 | TRCN0000054555 | CCGGGCCCTCATAAAGGCTGCACAACTCGAGTTGTGCAGCCTTTATGAGGGCTTTTTG |
| shYy1_4 | TRCN0000054556 | CCGGGTGGTTGAAGAGCAGATCATTCTCGAGAATGATCTGCTCTTCAACCACTTTTTG |
| shYy1_5 | TRCN0000054557 | CCGGCACATCTTAACACACGCTAAACTCGAGTTTAGCGTGTGTTAAGATGTGTTTTTG |
| shRuvbl1_1 | TRCN0000115241 | CCGGGCTGGAGATGTGATTTACATTCTCGAGAATGTAAATCACATCTCCAGCTTTTTG |
| shRuvbl1_2 | TRCN0000115243 | CCGGGCTGGCAAAGATCAATGGCAACTCGAGTTGCCATTGATCTTTGCCAGCTTTTTG |
| shRuvbl1_3 | TRCN0000115244 | CCGGGCCACAGAGTTTGACCTTGAACTCGAGTTCAAGGTCAAACTCTGTGGCTTTTTG |
| shRuvbl1_4 | TRCN0000115245 | CCGGGCAAGATATTCTGTCTATGATCTCGAGATCATAGACAGAATATCTTGCTTTTTG |
| shRuvbl2_1 | TRCN0000115256 | CCGGCCGAGAACAGATCAATGCAAACTCGAGTTTGCATTGATCTGTTCTCGGTTTTTG |
| shRuvbl2_2 | TRCN0000115257 | CCGGTGTGACAAGAATCGAGCGAATCTCGAGATTCGCTCGATTCTTGTCACATTTTTG |
| shRuvbl2_3 | TRCN0000115258 | CCGGCCTCCATGAGATTGACGTCATCTCGAGATGACGTCAATCTCATGGAGGTTTTTG |
| shRuvbl2_4 | TRCN0000115259 | CCGGCGACACACCATTCACAGCCATCTCGAGATGGCTGTGAATGGTGTGTCGTTTTTG |
| shRuvbl2_5 | TRCN0000115260 | CCGGGCCAGCCTGGTGTGCCGGAAACTCGAGTTTCCGGCACACCAGGCTGGCTTTTTG |
| shSmarca5_1 | TRCN0000084429 | CCGGCCGAGCAAATAGATTTGAGTACTCGAGTACTCAAATCTATTTGCTCGGTTTTTG |
| shSmarca5_2 | TRCN0000084430 | CCGGGTTTGGGAAAGACACTTCAAACTCGAGTTTGAAGTGTCTTTCCCAAACTTTTTG |
| shSmarca5_3 | TRCN0000084431 | CCGGCGTCGAATTAAAGCTGATGTTCTCGAGAACATCAGCTTTAATTCGACGTTTTTG |
| shSmarca5_4 | TRCN0000084432 | CCGGCCCTTTCATCAGCTAAGAATACTCGAGTATTCTTAGCTGATGAAAGGGTTTTTG |
| shSmarca5_5 | TRCN0000295773 | CCGGTCACTAACTGGTAGTTCTTTACTCGAGTAAAGAACTACCAGTTAGTGATTTTTG |
| shTet1_1 | TRCN0000341917 | CCGGTCTAACCAGTGTGCTAATATACTCGAGTATATTAGCACACTGGTTAGATTTTTG |
| shTet1_2 | TRCN0000341850 | CCGGCCTACGGGAAGCGACCATAATCTCGAGATTATGGTCGCTTCCCGTAGGTTTTTG |
| shTet1_3 | TRCN0000341847 | CCGGCAACTTGCATCCACGATTAATCTCGAGATTAATCGTGGATGCAAGTTGTTTTTG |
| shTet1_4 | TRCN0000341849 | CCGGCTAGCTATAGAGTATAGTAAACTCGAGTTTACTATACTCTATAGCTAGTTTTTG |
| shTet1_5 | TRCN0000341848 | CCGGTTTCAACTCCGACGTAAATATCTCGAGATATTTACGTCGGAGTTGAAATTTTTG |
| shTet2_1 | TRCN0000217530 | CCGGCTTGTACTGTATAGGCATAAGCTCGAGCTTATGCCTATACAGTACAAGTTTTTTG |
| shTet2_2 | TRCN0000192770 | CCGGCCAACTCATGGGTCAATTCTTCTCGAGAAGAATTGACCCATGAGTTGGTTTTTTG |
| shTet2_3 | TRCN0000201087 | CCGGCGCTGGACATTTGTCTTGAAACTCGAGTTTCAAGACAAATGTCCAGCGTTTTTTG |
| shTet2_4 | TRCN0000250893 | CCGGTCGTGCTTTGGCCAGATTAAACTCGAGTTTAATCTGGCCAAAGCACGATTTTTG |
| shTet2_5 | TRCN0000250895 | CCGGGAGCGTTCCTCAGTATCATTTCTCGAGAAATGATACTGAGGAACGCTCTTTTTG |
| shTrrap_1 | TRCN0000088534 | CCGGCCCAGCGCATTGAACTGCTTTCTCGAGAAAGCAGTTCAATGCGCTGGGTTTTTG |
| shTrrap_2 | TRCN0000088535 | CCGGCATCAAGATCATAAGCATCATCTCGAGATGATGCTTATGATCTTGATGTTTTTG |
| shTrrap_3 | TRCN0000088536 | CCGGCGCTGAACTTTACTCTCTGTTCTCGAGAACAGAGAGTAAAGTTCAGCGTTTTTG |
| shTrrap_4 | TRCN0000088537 | CCGGGTGTCAACTCAGTGTCCATTACTCGAGTAATGGACACTGAGTTGACACTTTTTG |
| shSf1_1 | TRCN0000109275 | CCGGCCCAAGACGAATATCCAGAAACTCGAGTTTCTGGATATTCGTCTTGGGTTTTTG |
| shSf1_2 | TRCN0000109276 | CCGGGCGTAAAGATGGTCAGATGTTCTCGAGAACATCTGACCATCTTTACGCTTTTTG |
| shSf1_3 | TRCN0000109277 | CCGGCCGATGGAACCAAGACACAATCTCGAGATTGTGTCTTGGTTCCATCGGTTTTTG |
| shSf1_4 | TRCN0000109278 | CCGGTGTAGCATCGAGTGTCTTCTTCTCGAGAAGAAGACACTCGATGCTACATTTTTG |
| shSf1_5 | TRCN0000109279 | CCGGGCACGGATGGATAAAGAATATCTCGAGATATTCTTTATCCATCCGTGCTTTTTG |
| shSf3a1_1 | TRCN0000109205 | CCGGGCCAGTCTACGGAATGAATATCTCGAGATATTCATTCCGTAGACTGGCTTTTTG |
| shSf3a1_2 | TRCN0000109206 | CCGGCCGAGTAGAATGGGCCAAATTCTCGAGAATTTGGCCCATTCTACTCGGTTTTTG |
| shSf3a1_3 | TRCN0000109207 | CCGGCGGAAGGACTATGATCCCAAACTCGAGTTTGGGATCATAGTCCTTCCGTTTTTG |
| shSf3a1_4 | TRCN0000109208 | CCGGCCAGGATCAATGTGGTACCTACTCGAGTAGGTACCACATTGATCCTGGTTTTTG |
| shSf3a1_5 | TRCN0000109209 | CCGGGCCTGAATTTGAAGCTAGGATCTCGAGATCCTAGCTTCAAATTCAGGCTTTTTG |
| shSf3b1_1 | TRCN0000109240 | CCGGCCTGCTTCAATCTGATGATATCTCGAGATATCATCAGATTGAAGCAGGTTTTTG |
| shSf3b1_2 | TRCN0000109241 | CCGGGCCCACTTTCTGATGAAGAATCTCGAGATTCTTCATCAGAAAGTGGGCTTTTTG |
| shSf3b1_3 | TRCN0000109242 | CCGGCGCAGCAGATATTGACCATAACTCGAGTTATGGTCAATATCTGCTGCGTTTTTG |
| shSf3b1_4 | TRCN0000109243 | CCGGGCCGAGAGATTATTTCTAATTCTCGAGAATTAGAAATAATCTCTCGGCTTTTTG |
| shSf3b1_5 | TRCN0000109244 | CCGGGCCTGCCTTAATGAATGAATACTCGAGTATTCATTCATTAAGGCAGGCTTTTTG |
| shU2af1_1 | TRCN0000123549 | CCGGCCTCTTGAACATTTACCGTAACTCGAGTTACGGTAAATGTTCAAGAGGTTTTTG |
| shU2af1_2 | TRCN0000123550 | CCGGCGACTTGAATAACCGTTGGTTCTCGAGAACCAACGGTTATTCAAGTCGTTTTTG |
| shU2af1_3 | TRCN0000123551 | CCGGCCAACCTTTAGCCAGACCATTCTCGAGAATGGTCTGGCTAAAGGTTGGTTTTTG |
| shU2af1_4 | TRCN0000123552 | CCGGGCCCTCTTGAACATTTACCGTCTCGAGACGGTAAATGTTCAAGAGGGCTTTTTG |
| shU2af1_5 | TRCN0000123553 | CCGGCCAGTAACTGACTTCAGGGAACTCGAGTTCCCTGAAGTCAGTTACTGGTTTTTG |
| shZrsr2_1 | TRCN0000126324 | CCGGCGAGAGATACTTACCAAGTAACTCGAGTTACTTGGTAAGTATCTCTCGTTTTTG |
| shZrsr2_2 | TRCN0000126325 | CCGGCCTTTACCCTAAGTAAGTTATCTCGAGATAACTTACTTAGGGTAAAGGTTTTTG |
| shZrsr2_3 | TRCN0000126326 | CCGGGAAGAACTAGAGAGAAAGTTACTCGAGTAACTTTCTCTCTAGTTCTTCTTTTTG |
| shZrsr2_4 | TRCN0000126327 | CCGGTGGTGCAATGACAGATACCTTCTCGAGAAGGTATCTGTCATTGCACCATTTTTG |
| shZrsr2_5 | TRCN0000126328 | CCGGTGGAAACACTAGTTTCCAAATCTCGAGATTTGGAAACTAGTGTTTCCATTTTTG |

**Table S2. List of Primers and Taqman Probes.**

**Names and sequences of primers for gene expression**

| Gene name | Sequence (Forward) | Sequence (Reverse) |
|---|---|---|
| *Gapdh* | AGGTCGGTGTGAACGGATTTG | TGTAGACCATGTAGTTGAGGTCA |
| *Myh6* | GCAGAACAGTAAAATTGAGGACG | CGCAGCTTCTCCACCTTAG |
| *Tnnt2* | CAGAGGAGGCCAACGTAGAAG | CTCCATCGGGGATCTTGGGT |
| *Actc1* | CTGGATTCTGGCGATGGTGTA | CGGACAATTTCACGTTCAGCA |
| *Actn2* | TGGCACCCAGATCGAGAAC | GTGGAACCGCATTTTTCCCC |
| *Col1a1* | GCTCCTCTTAGGGGCCACT | CCACGTCTCACCATTGGGG |
| *Sf3a1* | AAGAAGAAGCATCGTCAAAGGAG | GCATGGTAAGGGTCATTGGGA |
| *Sf3b1* | GTGGGCCTTGATTCCACAGG | GGCTTCTTCTGACCGAGCAA |
| *Yy1* | TCAGACCCTAAGCAACTGGC | GTGCAGCCTTTATGAGGGCAA |
| *Smarca5* | GACACCGAGATGGAGGAAGTA | CGAACAGCTCTGTCTGCTTTA |
| *Mll2* | TAAAACCGCCTATGGAGGACC | GGCGCATCAATGGGGATCT |
| *Mll5* | TTCAGACCTCCTGTAGAGAGC | AAAAGTCCTCGCATCAACACA |
| *Tet1* | ACACAGTGGTGCTAATGCAG | AGCATGAACGGGAGAATCGG |
| *Ctcf* | TTTGGATCGTCACATGAAAAGCC | ACAAAGGCCATATCGCAGTCT |
| *Mll1* | ATGAGCAGTTCTTAGGTTTTGGC | CTCCCGCGAGGTTTTCGAG |
| *Kdm5d* | TTCGGTCCCACTACGAACG | TTCCTCGTCTACTGTAGCAACT |
| *Phf12* | GGGGCTGATGGAGCAAATCC | CCACCTTCCTTGCAGCTATCG |
| *Sin3b* | GTGGAGGACGCTCTCACCTA | CGATGCTCTGGCTTTTGAACT |
| *Bcorl1* | CGCATGTGTGGCATCAACG | TCCCAACATAGTCCATTTTTGGC |
| *Ruvbl2* | GCCACCACCAAAGTCCCTG | CCCTTCTCGGATCATCTCCAG |
| *Tet3* | TGCGATTGTGTCGAACAAATAGT | TCCATACCGATCCTCCATGAG |
| *Rad21* | GCCCATGTATTTGAGTGCAACT | ATAGATGCGGACTACTCCCAG |
| *Sf1* | GAGAGTTCCGTACCCGCAAAA | CTCTGGGTCCAATTAGGAGCC |
| *Trrap* | CAACGCCCAGATCGGACTG | GAGGCTTTTGTAACAGGGCAG |
| *U2af1* | TCGGAGCATGTCGTCATGG | GGAAGAGTTTTGAGGGTTACGG |
| *Kdm5c* | GAGGCCCAGACAAGAGTGAAA | TTGGGAATCTTTAAGGATGAGCC |
| *Phf23* | CACATCATTGACGCATACTTCCC | TTTCGGTCCTTCTTTCGAGGC |
| *Phf23* | CACATCATTGACGCATACTTCCC | TTTCGGTCCTTCTTTCGAGGC |
| *Stag2* | CTACAAGCATGACCGGGACAT | GCCGTACTAACACACCAATGAAC |
| *Ruvbl1* | AGCTGGGCAGTAAAGTCCCT | CCTCCCCTTCATAAACCTCCT |
| *Bcor* | CTTTCTGCAACCCCTCTGTATG | AAGCGTCGCCATCATTCACA |

**Applied Biosystems Taqman probes**

| **Gene** | **Vendor** | **Product** |
|---|---|---|
| *Gapdh* | applied biosystems | Mm99999915_g1 |
| *Scn5a* | applied biosystems | Mm01342518_m1 |
| *Kcnj2* | applied biosystems | Mm00434616_m1 |
| *Cacna1c* | applied biosystems | Mm00437917_m1 |
| *Col1a2* | applied biosystems | Mm00483888_m1 |
| *Col3a1* | applied biosystems | Mm01254476_m1 |
| *Postn* | applied biosystems | Mm00450111_m1 |
